# Simultaneously Assessed Three-Dimensional Speckle-Tracking Echocardiography-Derived Left Ventricular and Left Atrial Volumes Are Associated in Healthy Adults—Findings from the MAGYAR-Healthy Study

**DOI:** 10.3390/jcm12124153

**Published:** 2023-06-20

**Authors:** Attila Nemes, Árpád Kormányos, Zoltán Ruzsa, Alexandru Achim, Nóra Ambrus, Csaba Lengyel

**Affiliations:** Department of Medicine, Albert Szent-Györgyi Medical School, University of Szeged, 6725 Szeged, Hungary; kormanyos.arpad@med.u-szeged.hu (Á.K.); zruzsa25@gmail.com (Z.R.); dr.alex.achim@gmail.com (A.A.); ambrus.nora@med.u-szeged.hu (N.A.); lecs@in1st.szote.u-szeged.hu (C.L.)

**Keywords:** left ventricular, left atrial, volume, three-dimensional, speckle-tracking, echocardiography

## Abstract

Introduction: The left atrium (LA) has a significant role in regulating blood flow from veins to the left ventricle (LV). LV performance is affected by several factors including preload, which is partly, but highly, dependent on LA volumes. The aim of the present study is to perform simultaneous assessment of LA and LV volume changes during the cardiac cycle in healthy circumstances. Therefore, LA and LV volumes and volume-based functional properties were determined in healthy adults, and the associations of these parameters were examined. Methods: The present study consists of 164 healthy adults (age: 33.0 ± 12.3 years, 82 males) being in sinus rhythm. All subjects have undergone complete two-dimensional Doppler echocardiography with three-dimensional speckle-tracking echocardiography (3DSTE). Results: Increased end-systolic maximum LA volume was associated with higher LV volumes and reduced LV ejection fraction. Very high early pre-atrial contraction and late diastolic LA volumes were associated with increased LV volumes, reduced LV ejection fraction and increased LV mass. Increased LA volumes were associated with increased LV mass. Higher LV volumes were associated with tendentiously higher LA volumes. Higher LV end-diastolic volume was associated with tendentiously higher all LA stroke volumes (SVs) and total and active LA emptying fractions (EFs). Higher LV end-systolic volume was associated with tendentiously higher all LA SVs but preserved all LA EFs. Conclusions: 3DSTE is capable of simultaneous assessment of LA and LV volumes and volume-based functional properties for (patho)physiologic studies. Moreover, 3DSTE-derived LV and LA volumes and functional properties show strong associations.

## 1. Introduction

The left atrium (LA) has a significant role in regulating blood flow from veins to the left ventricle (LV) [1]. LV performance is affected by several factors such as preload, afterload, intrinsic or extrinsic inotropic effects, LV geometry, etc. [2]. LV preload, which refers to the amount of blood already in LV when it is ready to pump it out, is partly, but highly, dependent on LA volumes [3]. Three-dimensional speckle-tracking echocardiography (3DSTE) is a novel imaging method with the ability to perform simultaneous LA and LV volumetric measurements using the same 3D datasets, which seems to be optimal for physiologic studies [4,5,6,7,8]. The present study aims to quantify 3DSTE-derived LA and LV volumes during the cardiac cycle in the same subjects to compare their associations in healthy circumstances. 

## 2. Materials and Methods

### 2.1. Subject Population

The present study consists of 164 healthy adults (age: 33.0 ± 12.3 years, 82 males), who were in sinus rhythm. All volunteers were recruited in a screening program between 2011 and 2015 during which physical examination, laboratory tests, standard 12-lead electrocardiography (ECG), two-dimensional Doppler echocardiography (2DE), and 3DSTE were performed. Subjects showing negative results were selected for this study. None of them used drugs or had a known disease, pathological state, or clinical condition that could affect the results. Because of the known negative effects of smoking on cardiac function, all subjects were non-smokers [9]. The present retrospective cohort study is part of the Motion Analysis of the heart and Great vessels bY three-dimensionAl speckle-tRacking echocardiography in Healthy subjects (MAGYAR-Healthy) Study, which has been organized at the University of Szeged partly for the purpose of examining physiologic correlations (‘Magyar’ means ‘Hungarian’ in the Hungarian language). The study was conducted in accordance with the Declaration of Helsinki (as revised in 2013). The study was approved by the Institutional and Regional Human Biomedical Research Committee of University of Szeged, Hungary (No.: 71/2011 and updated versions), and participants gave informed consent.

### 2.2. Routine 2DE

Complete 2DE was completed in all cases including chamber quantifications, Doppler assessments of the degree of valvular regurgitations and stenoses, and determination of early (E) and late (A) diastolic mitral inflow velocities and their ratio (E/A). For these aims, a Toshiba Artida^TM^ echocardiographic tool (Toshiba Medical Systems, Tokyo, Japan) with a PST-30BT (1–5 MHz) phased-array transducer was used. All 2DE studies were carried out in accordance with recent guidelines and practices [10]. 

### 2.3. 3DSTE-Derived Data Acquisition

The same Toshiba Artida^TM^ echocardiographic tool (Toshiba Medical Systems, Tokyo, Japan) was used for 3DSTE-derived data acquisitions after replacing the transducer with a PST-25SX matrix transducer [4,5,6,7]. Subjects were asked to lie in left lateral position, and then the transducer was positioned on his/her chest in an optimal location for data acquisition from the apical window. During breath hold, 6 subvolumes were acquired within 6 cardiac cycles. These subvolumes were merged automatically into a full volume ‘pyramid-shape’ 3D echocardiographic dataset for later analysis by a vendor-provided 3D Wall Motion Tracking software version 2.7 (Ultra Extend, Toshiba Medical Systems, Tokyo, Japan). The study was completed by a single observer (ÁK).

### 2.4. LV Volume Measurement with 3DSTE

**Figure 1:**
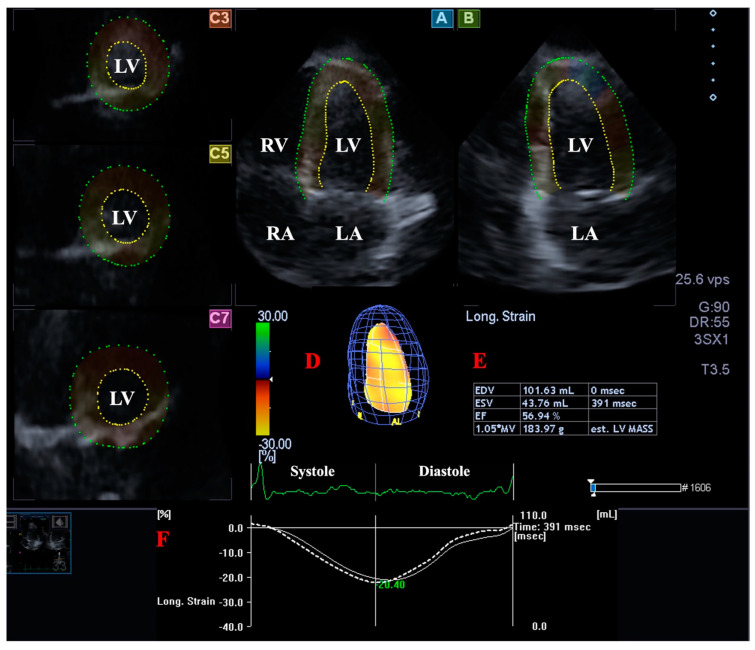
Three-dimensional (3D) speckle-tracking echocardiography-derived left ventricular (LV) volumetric analysis. Apical four-chamber view (A); apical two-chamber view (B); short-axis view at basal (C3), midventricular (C5), and apical LV level (C7) are demonstrated together with a 3D cast of the LV (D) and calculated LV volumetric data (E). Time-global LV longitudinal strain curve (white line) and time-LV volume change curve (dashed white line) are shown as well (F). Abbreviations. LA: left atrium, LV: left ventricle, RA: right atrium, RV: right ventricle, EDV: end-diastolic volume, ESV: end-systolic volume, EF: ejection fraction.

Using the above mentioned software, data were presented in optimal apical longitudinal 4-chamber (AP4CH) and 2-chamber (AP2CH) views, and basal, midventricular, and apical cross-sectional views focused on the LV. Following definition of mitral annular (MA)–LV edges and endocardial surface of the LV apex, sequential analysis was performed proceeding into the creation of a virtual 3D LV cast (Figure 1). Using this LV model, end-systolic (ESV) and end-diastolic (EDV) LV volumes, LV ejection fraction (E-Fr), and LV mass were determined [4,5,6,7,11]. 

### 2.5. LA Volume Measurement with 3DSTE

**Figure 2:**
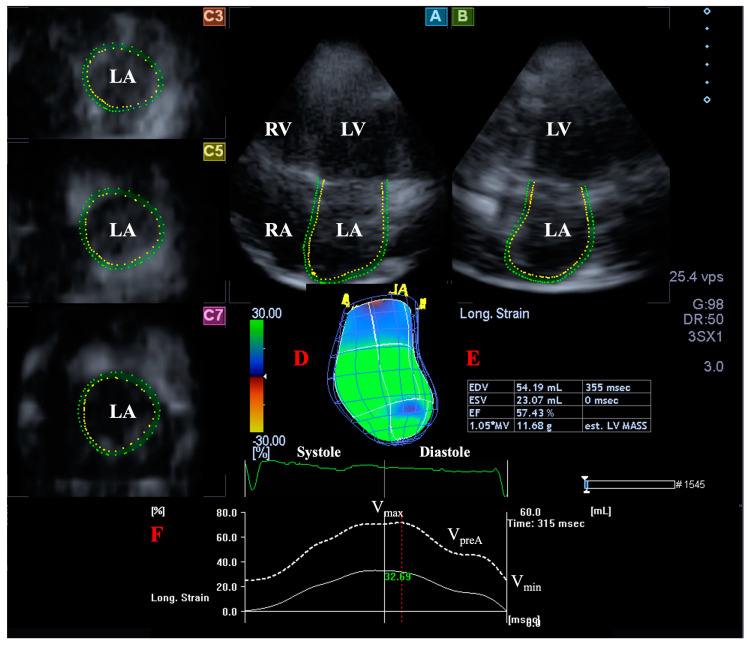
Three-dimensional (3D) speckle-tracking echocardiography-derived left atrial (LA) volumetric analysis. Apical four-chamber view (A); apical two-chamber view (B); short-axis view at basal (C3), midatrial (C5), and superior left atrial level (C7) are demonstrated together with a 3D cast of the LA (D) and calculated LA volumetric data (E). Time-global LA longitudinal strain change (white line) and time-LA volume change curve (dashed white line) are shown as well (F). Abbreviations. LA: left atrium, LV: left ventricle, RA: right atrium, RV: right ventricle, EDV: end-diastolic volume, ESV: end-systolic volume, EF: ejection fraction, V_max_: end-systolic maximum LA volume, V_preA_: early diastolic pre-atrial contraction LA volume: V_min_, end-diastolic minimum LA volume.

A similar LA analysis was also performed on images focusing on the LA in longitudinal AP4CH and AP2CH views and 3 short-axis views in basal, midatrial, and superior LA regions [1,12]. A 3D model of the LA was created as an analogy for LV assessments: reference points were set in AP4CH and AP2CH starting at the lateral edge of the MA–LA going toward the LA apex to the edge of the septal MA–LA, and then sequential analysis was performed. According to recent practices, LA appendage and pulmonary veins were excluded from the measurements (Figure 2). The following LA volumes were determined [4,5,6,7,8,13]:

- End-systolic maximum LA volume (V_max_), measured just before mitral valve opening.
- LA volume before atrial contraction in early diastole (V_preA_), measured at the time of P wave on ECG.
- Late diastolic minimum LA volume (V_min_), measured just before mitral valve closure.

Using LA volumes, different phases of LA function were featured using the following parameters [8,13]:

*End-systolic reservoir LA function*

- TASV—LA total stroke volume, calculated by V_max_ − V_min_.
- TAEF—LA total emptying fraction, calculated by total SV/V_max_.

*Early diastolic conduit LA function*

- PASV—LA passive stroke volume, calculated by V_max_ − V_preA_.
- PAEF—LA passive emptying fraction, calculated by passive SV/V_max_.

*Late diastolic booster pump (active contraction) LA function*

- AASV—LA active stroke volume, calculated by V_preA_ − V_min_.
- AAEF—LA active emptying fraction, calculated by active SV/V_preA_.

### 2.6. Statistical Analysis

Continuous variables are presented as mean ± standard deviation (SD), while number/percentage format is used for categorical data. Results are considered statistically significant when *p* < 0.05. Shapiro–Wilks test was used to assess normality of distribution and Levene’s test to test homogeneity of variances. If datasets were proven to be normally distributed, Student’s *t*-test was used; if they turned out to be non-normally distributed, Mann–Whitney–Wilcoxon test was performed. Analysis of variance (ANOVA) tests were used for group comparisons where appropriate. Pearson’s correlation coefficients were calculated for featuring correlations. SPSS software version 22 (SPSS Inc., Chicago, IL, USA) was used during statistical analyses.

## 3. Results

### 3.1. Clinical and 2DE Data

The systolic and diastolic blood pressures and heart rate proved to be 119 ± 4 mmHg, 78 ± 4 mmHg, and 71 ± 3 1/s, respectively. The mean height, weight, and calculated body surface area were 171.1 ± 9.3 cm, 73.5 ± 10.8 kg, and 1.87 ± 0.21 kg/m^2^, respectively. None of the participants had larger than grade 1 valvular regurgitation or significant stenosis on any valves. Routine 2DE data were in normal ranges: LA diameter measured in parasternal long-axis view (36.7 ± 4.0 mm), LV end-diastolic diameter (48.1 ± 3.7 mm) and volume (107.0 ± 23.0 mL), LV end-systolic diameter (40.5 ± 25.6 mm) and volume (36.6 ± 9.3 mL), interventricular septum (9.0 ± 1.6 mm), LV posterior wall (9.1 ± 1.6 mm), and LV E-Fr (65.9 ± 4.9%). Mean E/A proved to be 1.36 ± 0.38. 

### 3.2. Classification of Subjects

3DSTE-derived LV and LA volumes and their indexed counterparts are presented in Table 1. LV-EDV, LV-ESV, and V_max_, V_preA_, and V_min_ were measured and expressed as mean ± SD. Study cases were classified into 3 groups on the basis of these values: subjects with values within the normal range and subjects with lower (62.2 mL, 25.8 mL, 27.9 mL, 15.9 mL, and 11.3 mL, respectively) and higher (109.0 mL, 46.6 mL, 54.0 mL, 39.3 mL, and 27.3 mL, respectively) values. 

### 3.3. Higher LA Volumes vs. LV Parameters

Increased V_max_ was associated with higher LV volumes, indexed LV volumes, and reduced LV-E-Fr. Very high diastolic V_preA_ and V_min_ were associated with increased LV volumes, indexed LV volumes, reduced LV-EF, and increased LV mass. Increased LA volumes were associated with increased LV mass (Table 2).

### 3.4. Higher LA Volumes vs. LA Functional Properties

Higher end-systolic V_max_ was associated with higher diastolic V_preA_, V_min_, and their indexed counterpart, and vice versa. Higher V_max_ was associated with increased all LA-SVs and AAEF, preserved TAEF, and reduced PAEF. Higher V_preA_ was associated with increased TASV, AASV, and AAEF; preserved PASV; and reduced TAEF and PAEF. Higher V_min_ was associated with increased TASV and AASV, preserved PASV, and reduced all LA-EFs (Table 2).

### 3.5. Higher LV Volumes vs. LA Parameters

Higher LV volumes were associated with tendentiously higher LA volumes and their indexed counterpart. Higher LV-EDV was associated with tendentiously higher all LA-SVs, TAEF, and AAEF, but preserved PAEF. Higher LV-ESV was associated with tendentiously higher all LA-SVs, but preserved all LA-EFs (Table 3). 

### 3.6. Higher LV Volumes vs. LV-E-Fr

Higher LV-EDV was associated with higher LV-ESV and indexed-LV-ESV, preserved LV-EF, and, simultaneously, increased LV mass. Higher LV-ESV was associated with higher LV-EDV and indexed LV-EDV, tendentiously reduced LV-E-Fr, and, simultaneously increased LV mass (Table 3). 

### 3.7. Correlations

Direct correlations between LV and LA volumes and volume-based functional properties could not be detected.

## 4. Discussion

The primary function of the LV is to deliver blood through the body in systole via contraction of the myocardial walls [3]. For this aim, cardiac output, which is calculated as the product of the amount of blood that is pumped out during LV contraction (called the LV stroke volume) and the heart rate, should be maintained. LV-SV is the net difference between LV-EDV and LV-ESV. In clinical practice, LV-E-Fr, which is the ratio of SV and LV-EDV, is considered to be a significant predictor of future events and outcome [3]. The most important factors affecting the cardiac output are preload, afterload, and contractility [2]. In diastole, the LV is loaded from the LA, higher volumes increase contractility via the Frank–Starling mechanism while the preload volume lengthens the myocyte sarcomere length closer to the optimal overlap of actin and myosin [14]. According to these facts, LA has a significant role in regulating LV function via preload volumes. In other words, LA acts like a reservoir in systole, having the largest volume in this phase of cardiac function [1]. In early diastole, the LV fills from the LA via the mitral valve and annulus, while the LA works like a conduit forwarding blood from veins to the LV. At the end of diastole, it has a booster pump function, when it actively contracts, helping LV loading and concomitant LA emptying [1,3]. 

As a result of significant developments in non-invasive cardiovascular imaging, mainly in novel echocardiographic techniques, detailed volumetric and functional analysis of cardiac chambers has become part of daily routine. For example, 3DSTE facilitates simultaneous determination of LA and LV volumes and volume-based functional properties (LA-SVs, LA-EFs, and LV-E-Fr) using the same acquired 3D echocardiographic dataset [4,5,6,7,8]. This sort of approach provides an opportunity for (patho)physiologic studies comparing 3DSTE-derived parameters with each other. Further, 3DSTE is validated for LA [12,15,16] and LV [17,18] volumetric assessments with good reliability [19], and normal reference values are also determined [11,13]. 

To the best of the authors’ knowledge, this is the first study to analyze 3DSTE-derived LA and LV volumes and functional properties at the same time, demonstrating their associations in healthy adults with normofrequent sinus rhythm. Higher LA volumes were associated with elevated LV volumes, reduced LV function represented by LV-E-Fr, and increased LV mass. Conversely, higher LV volumes were associated with tendentiously higher LA volumes and increased LV mass. While higher LV-EDV was associated with preserved LV-E-Fr, higher LV-ESV was associated with a tendentious reduction in LV pumping function. These associations reflect complex adaptations between LA and LV volumes, which have effects on their functional properties as well. 

These results can be interpreted in the context of previous findings, which demonstrated strong associations between LV rotational mechanics, a significant contributor to LV function and LA volumes in healthy adults, enabling a more detailed focus on understanding the complexity of the volumetric and functional relationship between the LV and LA [20]. It can be demonstrated that while counterclockwise apical LV rotation was not associated with increasing LA volumes, basal LV rotation was highest in the case of the highest V_max_ and V_preA_. While reduced diastolic LA volumes were seen in the case of increased apical LV rotation, the highest basal LV rotation was associated with increased diastolic LA volumes. These findings suggest the high dependence of LV rotational mechanics on LA volumes with respect to the cardiac cycle [20].

The clinical implication of this study is that clinicians should be aware of the fact that there are strong correlations between LA and LV volumes even in healthy circumstances. Moreover, changes in volumes are associated with changes in functional properties and LV mass as well. However, more detailed clinical studies are warranted with recent cardiovascular imaging techniques for a better understanding of these (patho)physiological relationships and adaptations, even in non-healthy circumstances.

### Limitations Section

Several important limitations have arisen; the most important ones are listed below:

- Although we tried to include completely healthy cases in the study, it cannot be 100% ruled out that someone did not have an undiscovered disease or pathological state.
- 3DSTE-derived image quality is worse as compared to that of 2D echocardiography because of physical reasons, which could significantly affect the findings [4,5,6,7].
- The main advantage of 3DSTE is featuring myocardial contractility using myocardial strains. However, the present study did not aim for a comparison of such parameters, and only volumetric analyses were performed [4,5,6,7].

## 5. Conclusions

3DSTE is capable of simultaneous assessment of LA and LV volumes and volume-based functional properties for (patho)physiologic studies. Moreover, 3DSTE-derived LV and LA volumes and functional properties show strong associations. 

## Figures and Tables

**Table 1:** Three-dimensional speckle-tracking echocardiography-derived left atrial and left ventricular volumes and functional properties.

Parameters	Measures
Left atrial volumes and functional properties	
Maximum left atrial volume (V_max_, mL)	40.9 ± 13.0
Indexed maximum left atrial volume (indexed-V_max_, mL/m^2^)	22.4 ± 6.1
Pre-atrial contraction left atrial volume (V_preA_, mL)	27.6 ± 11.7
Indexed pre-atrial contraction left atrial volume (indexed-V_preA_, mL/m^2^)	15.2 ± 5.7
Minimum left atrial volume (V_min_, mL)	19.3 ± 8.0
Indexed minimum left atrial volume (indexed-V_min_, mL/m^2^)	10.5 ± 3.9
Total atrial stroke volume (TASV, mL)	21.5 ± 8.2
Total atrial emptying fraction (TAEF, %)	52.7 ± 11.9
Passive atrial stroke volume (PASV, mL)	13.2 ± 5.7
Passive atrial emptying fraction (PAEF, %)	33.4 ± 12.6
Active atrial stroke volume (AASV, mL)	8.3 ± 5.8
Active atrial emptying fraction (AAEF, %)	28.9 ± 12.0
Left ventricular volumes	
End-diastolic left ventricular volume (LV-EDV, mL)	86.1 ± 22.9
Indexed end-diastolic left ventricular volume (indexed-LV-EDV, mL/m^2^)	47.0 ± 12.7
End-systolic left ventricular volume (LV-ESV, mL)	36.2 ± 10.4
Indexed end-systolic left ventricular volume (indexed-LV-ESV, mL/m^2^)	19.8 ± 5.9
Left ventricular ejection fraction (LV-EF, %)	58.2 ± 5.6
Left ventricular mass (g)	158.8 ± 32.2

**Table 2:** Left ventricular and left atrial volumes in different left atrial volume groups.

	V_max_ < 27.9 mL (n = 23)	27.9 mL ≤ V_max_ ≤ 53.9 mL (n = 116)	54 mL < V_max_ (n = 25)	V_preA_ < 15.9 mL (n = 17)	15.9 mL ≤ V_preA_ ≤ 39.3 mL (n = 124)	39.3 mL < V_preA_ (n = 23)	V_min_ < 11.3 mL (n = 23)	11.2 mL ≤ V_min_ ≤ 27.3 mL (n = 118)	27.3 mL < V_min_ (n = 23)
Left atrial parameters									
V_max_ (mL)	23.7 ± 3.6	39.1 ± 6.2 *	64.8 ± 7.2 */**	25.8 ± 6.0	38.8 ± 8.5 †	63.2 ± 9.8 †/††	26.5 ± 5.4	39.7 ± 9.1 ‡	61.2 ± 11.1 ‡/‡‡
V_max_-indexed (mL/BSA)	12.7 ± 2.0	20.8 ± 3.5 *	34.7 ± 4.0 */**	13.8 ± 3.4	20.7 ± 4.7 †	34.0 ± 5.5 †/††	14.2 ± 3.0	21.1 ± 5.0 ‡	32.2 ± 6.2 ‡/‡‡
V_preA_ (mL)	16.0 ± 4.3	25.7 ± 6.9 *	47.3 ± 11.3 */**	12.9 ± 2.6	25.4 ± 5.9 †	50.4 ± 8.4 †/††	14.6 ± 3.4	26.2 ± 7.5 ‡	47.8 ± 9.3 ‡/‡‡
V_max_-indexed (mL/BSA)	8.5 ± 2.4	13.8 ± 3.9 *	25.1 ± 6.3 */**	6.8 ± 1.4	13.5 ± 3.3 †	26.8 ± 4.6 †/††	7.7 ± 1.9	14.1 ± 4.1 ‡	25.6 ± 5.3 ‡/‡‡
V_min_ (mL)	11.9 ± 3.8	18.3 ± 5.6 *	31.0 ± 8.7 */**	9.2 ± 1.9	18.2 ± 4.9 †	32.9 ± 7.9 †/††	9.2 ± 1.7	18.3 ± 4.0 ‡	34.6 ± 5.8 ‡/‡‡
V_max_-indexed (mL/BSA)	6.4 ± 2.1	9.7 ± 3.2 *	16.5 ± 4.9 */**	4.9 ± 1.1	9.6 ± 2.8 †	17.5 ± 4.4 †/††	4.9 ±1.0	9.7 ± 2.4 ‡	18.4 ± 3.3 ‡/‡‡
TASV (mL)	11.8 ± 3.2	20.8 ± 5.4 *	33.8 ± 7.5 */**	16.6 ± 5.1	20.6 ± 7.0 †	30.3 ± 10.0 †/††	17.3 ± 4.6	21.3 ± 8.1 ‡	26.6 ± 9.2 ‡/‡‡
TAEF (%)	50.0 ± 12.9	53.3 ± 11.9	52.3 ± 11.5	63.3 ± 8.1	42.3 ± 11.5 †	47.3 ± 12.2 †	64.5 ± 7.3	52.4 ± 11.1 ‡	42.5 ± 9.7 ‡/‡‡
PASV (mL)	7.7 ± 3.0	13.4 ± 4.8 *	17.6 ± 7.0 */**	12.9 ± 4.4	13.4 ± 5.8	12.8 ± 5.9	11.9 ± 4.4	13.4 ± 5.6	13.5 ± 6.9
PAEF (%)	33.1 ± 13.2	34.6 ± 12.2	27.7 ± 12.4 **	48.8 ± 8.8	33.7 ± 11.0 †	20.0 ± 7.9 †/††	44.3 ± 11.5	33.6 ± 11.2 ‡	21.4 ± 9.7 ‡/‡‡
AASV (mL)	4.1 ± 3.0	7.4 ± 3.6 *	16.2 ± 8.8 */**	3.8 ± 2.1	7.2 ± 3.4 †	17.4 ± 8.6 †/††	5.4 ± 3.2	7.9 ± 5.6 ‡	13.2 ± 6.2 ‡/‡‡
AAEF (%)	24.7 ± 15.0	28.7 ± 10.7	33.5 ± 13.6 *	27.9 ± 13.2	28.1 ± 11.2	34.0 ± 14.2 ††	34.7 ± 14.3	28.2 ± 11.8 ‡	26.7 ± 8.7 ‡
Left ventricular parameters									
LV-EDV (mL)	74.9 ± 16.0	84.6 ± 20.2 *	103.2 ± 30.7 */**	86.0 ± 15.7	83.2 ± 21.1	101.9 ± 30.3 ††	82.2 ± 15.3	84.6 ± 21.5	97.3 ± 32.5 ‡/‡‡
LV-EDV-indexed (mL/BSA)	39.8 ± 8.3	44.8 ± 11.0 *	55.2 ± 16.9 */**	46.0 ± 8.7	44.2 ± 11.5	54.2 ± 16.7 ††	43.3 ± 8.4	45.0 ± 11.9	51.8 ± 17.9 ‡/‡‡
LV-ESV (mL)	29.8 ± 8.1	35.9 ± 9.2 *	43.6 ± 13.4 */**	35.2 ± 9.8	34.8 ± 9.5	44.4 ± 12.3 †/††	33.9 ± 8.8	35.3 ± 9.7	43.1 ± 13.1 ‡/‡‡
LV-ESV-indexed (mL/BSA)	15.9 ± 4.3	19.2 ± 5.2 *	23.4 ± 7.4 */**	18.9 ± 5.4	18.6 ± 5.2	24.0 ± 6.8 †/††	17.8 ± 4.9	18.7 ± 5.6	22.8 ± 7.3 ‡/‡‡
LV-EF (%)	60.4 ± 5.6	57.9 ± 5.5 *	57.5 ± 5.9	59.3 ± 6.3	58.5 ± 5.3	55.7 ± 6.4 ††	58.9 ± 5.5	58.6 ± 5.6	55.3 ± 5.1 ‡/‡‡
LV mass (g)	139.0 ± 29.4	156.8 ± 29.4 *	186.4 ± 30.1 */**	142.7 ± 33.7	155.2 ± 29.9	189.8 ± 23.5 †/††	136.2 ± 30.1	158.4 ± 30.0 ‡	182.2 ± 29.7 ‡/‡‡

* *p* < 0.05 vs. V_max_ < 27.9 ml; ** *p* < 0.05 vs. 27.9 mL ≤ V_max_ ≤ 53.9 mL; † *p* < 0.05 vs. V_preA_ < 15.9 mL; †† *p* < 0.05 vs. 15.9 mL ≤ V_preA_ ≤ 39.3 mL; ‡ *p* < 0.05 vs. V_min_ < 11.3 mL; ‡‡ *p* < 0.05 vs. 11.2 mL ≤ V_min_ ≤ 27.3 mL.

**Table 3:** Left ventricular and left atrial volumes in different left ventricular volume groups.

	LV-EDV ≤ 62.2 mL (n = 19)	63.2 mL < LV-EDV < 109.0 mL (n = 127)	109.0 mL ≤ LV-EDV (n = 18)	LV-ESV ≤ 25.8 mL (n = 18)	25.8 mL < LV-ESV < 46.6 mL (n = 118)	46.6 mL ≤ LV-ESV (n = 23)
Left atrial parameters						
V_max_ (mL)	33.1 ± 9.9	40.7 ± 12.6 *	50.2 ± 13.4 */**	33.5 ± 12.0	40.5 ± 11.7	49.8 ± 15.5 †/††
V_max_-indexed (mL/m^2^)	17.7 ± 5.5	21.6 ± 6.9 *	26.8 ± 7.5 */**	17.7 ± 6.8	21.7 ± 12.3	26.6 ± 6.5 †/††
V_preA_ (mL)	23.6 ± 9.1	27.3 ± 11.4	34.2 ± 13.8 */**	23.3 ± 9.6	27.2 ± 10.9 †	34.1 ± 14.7 †/††
V_preA_-indexed (mL/m^2^)	12.5 ± 5.1	14.5 ± 6.4	18.3 ± 7.6 */**	12.5 ± 5.4	14.6 ± 6.0 †	18.2 ± 8.3 †/††
V_min_ (mL)	17.5 ± 6.1	19.2 ± 8.2	22.5 ± 8.1 *	16.2 ± 5.3	19.1 ± 7.7	23.5 ± 10.4 †/††
V_min_-indexed (mL/m^2^)	9.4 ± 3.4	10.2 ± 4.6	12.0 ± 4.6 *	8.8 ± 3.1	10.2 ± 4.3	12.6 ± 5.8 †/††
TASV (mL)	15.6 ± 5.7	21.5 ± 7.7 *	27.7 ± 9.6 */**	17.3 ± 10.2	21.4 ± 7.2 †	26.3 ± 8.7 †/††
TAEF (%)	46.9 ± 10.0	53.3 ± 12.1 *	55.0 ± 11.6 *	49.5 ± 13.2	53.2 ± 11.7	53.7 ± 11.7
PASV (mL)	9.4 ± 3.7	13.4 ± 5.5 *	16.1 ± 6.6 *	10.3 ± 4.8	13.3 ± 5.5 †	15.6 ± 6.0 †
PAEF (%)	29.6 ± 10.8	33.9 ± 12.7	33.3 ± 13.5	31.0 ± 11.7	33.9 ± 12.7	33.2 ± 13.0
AASV (mL)	6.1 ± 4.3	8.1 ± 5.3	11.6 ± 8.9 */**	7.1 ± 7.0	8.1 ± 5.5	10.6 ± 5.9 †
AAEF (%)	24.3 ± 10.4	29.2 ± 11.9	32.0 ± 13.1 *	26.9 ± 14.1	28.9 ± 11.9	30.9 ± 10.0
Left ventricular parameters						
LV-EDV (mL)	55.9 ± 5.2	84.7 ± 12.0 *	131.4 ± 19.7 */**	63.9 ± 13.9	83.0 ± 14.6 †	124.1 ± 22.0 †/††
LV-EDV-indexed (mL/m^2^)	29.9 ± 3.0	45.5 ± 6.8 *	69.5 ± 11.0 */**	34.0 ± 7.7	44.4 ± 8.0 †	66.4 ± 12.0 †/††
LV-ESV (mL)	23.2 ± 5.1	35.4 ± 6.7 *	56.0 ± 8.8 */**	21.9 ± 3.2	35.4 ± 5.5 †	54.6 ± 8.1 †/††
LV-ESV-indexed (mL/m^2^)	12.4 ± 2.9	18.9 ± 3.7 *	29.8 ± 4.9 */**	11.6 ± 1.8	18.7 ± 3.1 †	29.2 ± 4.5 †/††
LV-EF (%)	59.1 ± 7.9	58.2 ± 5.5	57.2 ± 4.1	65.2 ± 6.1	57.3 ± 4.8 †	55.7 ± 3.8 †
LV mass (g)	132.6 ± 23.1	157.4 ± 29.0 *	196.1 ± 29.4 */**	133.2 ± 25.5	157.3 ± 28.5 †	19.2 ± 28.8 †/††

* *p* < 0.05 vs. LV-EDV ≤ 62.2 mL; ** *p* < 0.05 vs. 63.2 mL < LV-EDV < 109.0 mL; † *p* < 0.05 vs. LV-ESV ≤ 25.8 mL; †† *p* < 0.05 vs. 25.8 mL < LV-ESV < 46.6 mL.

## Data Availability

All data are available.
